# Reflexivity and positionality applied to medical practice: a study on implicit gender bias with medical students in a Swiss university

**DOI:** 10.1186/s12939-024-02222-3

**Published:** 2024-07-01

**Authors:** Francesca Arena, Elisa Geiser, Silva Auer, Carole Clair, Joëlle Schwarz

**Affiliations:** 1 Institute for Ethics, History and Humanities, Faculty of Medicine, University of Geneva, iEH2 Geneva, Switzerland; 2 Center for Primary Care and Public Health, Unisanté, University of Lausanne, Lausanne, Switzerland

**Keywords:** Implicit bias, Gender, Positionality, Reflexivity, Masculinity, Medical education, Medical practice

## Abstract

**Background:**

An array of evidence shows how the presence of implicit bias in clinical encounters can negatively impact provider-patient communication, quality of care and ultimately contribute to health inequities. Reflexive practice has been explored as an approach to identify and address implicit bias in healthcare providers, including medical students. At the Lausanne School of Medicine, a clinically integrated module was introduced in 2019 to raise students’ awareness of gender bias in medical practice using a reflexivity and positionality approach. The purpose of this study is to describe the gender bias that were identified by medical students, analysing their types, places and modes of emergence during a clinical encounter. It further explores how positionality supported students’ reflection on the way in which social position modulates their relationship to patients.

**Methods:**

As part of the teaching activity, medical students individually reflected on gender bias in a specific clinical encounter by answering questions in their electronic portfolio. The questionnaire included a section on positionality. We qualitatively analysed the students’ assignments (*n*=76), applying a thematic analysis framework.

**Results:**

Medical students identified and described gender biases occurring at different moments of the clinical encounter (anamnesis (i.e. patient history), physical exam, differential diagnosis, final management). They causally associated these biases with wider social phenomena such as the gendered division of labour or stereotypes around sexuality and gender. Analysing students' reflections on how their position influenced their relationship with patients, we found that the suggested exercise revealed a major contradiction in the process of medical enculturation: the injunction to be neutral and objective erases the social and cultural context of patients and impedes an understanding of gender bias.

**Conclusion:**

Gender biases are present in the different steps of a clinical consultation and are rooted in broader gendered social representations. We further conclude that the tension between a quest for objectivity and the reality of social encounters should be made explicit to students, because it is constitutive of medical practice.

## Background

Medical practice is performed through the interaction of a physician with a patient. Whether such social interaction is a new encounter, or the two actors have encountered before, an unconscious ubiquitous assessment is processed by each actor based on the actor’s own social representations and on the social characteristics of the other actor such as age, gender, race, weight, accent. There is an array of evidence showing how the presence of implicit bias – associations that are made without the actor being aware of the process [1] – in clinical encounters can negatively impact provider-patient communication and trust relationship, quality of care and ultimately contributes to health inequities [1–5]. It has been observed for example that physicians tend to enquire around the family situation of female patients more than male patients, being influenced by the gendered view that family matters are women’s issues [6, 7]. Another example being the bias that may occur related to the weight of patients whereby the implicit unconscious thought is that overweight and obesity are a result of low willpower and individual responsibility [8]. Implicit bias is activated by situational cues – external features mostly – “silently exerting [their] influence on perception, memory and behaviour” [2]. In medicine, patients should be treated equally regardless of their gender for some aspects (e.g. asking about the family and professional context), but sometimes it is clinically justified to treat them differently, based on their specific needs (e.g. asking about hormonal contraceptive use in cis-women and trans-men when collecting information about medication). Implicit bias leads to unequal treatment in medical practice that is not clinically justified, but is based on perceived needs – unconsciously assumed from stereotypical social categorisation – that may not align with the needs of the individual.

Different approaches have been proposed and tested to tackle implicit bias in clinical practice. As suggested by Sabin, measures need to be implemented at two levels: the individual level to raise awareness of biases in general, and of one’s own biases; and the institutional level to propose, promote and monitor bias awareness raising activities, as well as skill building education in equality, diversity and inclusion (EDI) [9]. Another scoping review reported that among measures to reduce disparities in healthcare, raising clinician’s adherence to guidelines showed positive results [10]. The authors however reported that “in contrast to the wide research identifying gender bias in health care, few studies, so far, have described and evaluated interventions aimed to tackle this bias”. The few studies retrieved for the review emanated from specialised health care and hospitals, revealing a lack of studies addressing implicit bias in primary healthcare. Blair and colleagues propose a roadmap for future research on implicit bias in health care, calling for interventions to reduce their effects [2]. They place the possible interventions at the individual level (health care providers); at the level of persons (patients) discriminated by implicit bias through self-affirmation interventions; and last, at the team, clinic or delivery system level. Gonzales and colleagues offer tips to tackle implicit bias through teaching activities that include creating a safe environment, presenting the (cognitive) science of implicit bias and evidence of its influence on care, and suggest using approaches such as critical reflexive practice, which include skill-building activities in which participants explore and embrace their discomfort [11].

Reflexive practice (Table 1) has been explored as an approach to identify and address implicit bias in healthcare providers, including medical students [12]. At the Medical School of the University of Lausanne, Switzerland, a clinically integrated approach to raise students’ awareness on gender bias in medical practice was introduced in 2019, namely the Gender reflexivity project [7]. The approach was dual: 1st-year medical master students reflected in small groups on implicit gender bias identified during their clinical internships (group discussions); students individually brought their reflection further answering open questions on their personal student portfolio (individual reflections). Based on the positive assessment of this activity [7], the approach was further developed to include repeated group sessions with the same students, as well as new questionnaires to trigger a self-reflection on aspects of positionality and on how students’ social position may influence their current and future encounters with patients (Table 2).

**Table 1:** Definition of reflexive practice

Reflexive practice is the action of reflection, defined by Nguyen et al as “the process of engaging the self in *attentive, critical, exploratory and iterative *interactions with one’s *thoughts and actions*, and their underlying *conceptual frame*, with a *view to changing* them and with a *view on the change* itself.” [13]	

**Table 2:** Definition of positionality

Largely used in qualitative research in social sciences, positionality describes the researcher’s world view and the position they adopt to conduct their research in context. Researcher’s world view and position are “coloured by [their] values and beliefs that are shaped by their political allegiance, religious faith, gender, sexuality, historical and geographical location, ethnicity, race, social class, and status, (dis)abilities and so on” [14]. Applied to medical studies, we use positionality to explore how students’ identities and social positions influence professional identity formation, as well as medical practice through their encounters with patients.	

The purpose of this study is to describe the gender biases that were identified by medical students during their internship in the general practice outpatient clinic. It aims to analyse how students reflected on the manifestation of bias—their types, places and modes of emergence during a clinical encounter – and how they perceived them in terms of causes and consequences. It further explores how positionality supported students’ reflection on the way in which their social position modulates their relationship to patients. In the last section, we discuss how the reflexivity and positionality exercise with medical students raises a major tension. Namely a tension between the general process of becoming doctors – i.e. students’ enculturation into the medical world of neutrality and objectivity – and the exercise proposed of positioning oneself in an array of diversity and power that are rooted in social and subjective worlds. We conclude that such tension should be made explicit with students because it is constitutive of medical practice. Understanding of power dynamics and social interactions need to be re-injected in medical training in order to better reflect and prepare for (future) medical practice.

## Methods

In this study, we used material collected in the Gender reflexivity project between 2021 and 2022 and conducted a qualitative analysis of the students’ written reflections.

### Study setting and study material

Started in 2019, the Gender reflexivity project takes place in the general practice (GP) outpatient clinic at Unisanté (University Centre for Primary Care and Public Health), in Lausanne, Switzerland. The GP outpatient clinic is a public entity located in the centre of the city of Lausanne, attracting a diverse population in terms of socioeconomic status and health status. The project participants were 1st year Master medical students who underwent their internship in the GP outpatient clinic over a period of three to six weeks. During the internship, in addition to observing and partially conducting medical consultations, they were taught to present a clinical case through the structured clinical reasoning steps. The latter activity took place once a week where, in groups of five students, each student presented a clinical case encountered during the week to a chief resident (the teaching activity is coordinated by SA, a female senior physician). The Gender reflexivity project was integrated in this activity, whereby students were asked to reflect on potential gender bias influencing the specific clinical case presented, with the support and guidance of a gender medicine expert (a female general practitioner with expertise in gender medicine (EG) or a female health sociologist (JS)).

At the end of each week, students were asked to individually document a structured “reflection questionnaire” on their personal online portfolio. Three different reflection questionnaires were developed and used to gradually guide students towards more in-depth reflection on gender bias (Table 3).

**Table 3:** Incremental questions for reflection

Questionnaire 1	Describe the clinical case encountered and reflect on whether the steps of clinical reasoning would have differed if the patient was from the opposite sex What were the important points discussed during the group discussion? What were the most important elements that you have learned? What aspects were more difficult to address? What will you integrate into your clinical practice?
Questionnaire 2	Describe a clinical case encountered or discussed that presents gender bias Describe the potential gender biases that could occur in this clinical case described above In your opinion, are these biases attributable to clinical practice and/or to knowledge? In your opinion, what could be the factors and/or mechanisms that generate these practice/knowledge biases? In your opinion, what are the potential consequences of such bias for patients (overall management including the therapeutic relationship)? What could have been done to limit bias in the case presented (before – during – after the consultation)?
Questionnaire 3	Identify and briefly describe a clinical case where your practice may have been influenced by a gender bias or stereotype In your opinion, to what extent do the social characteristics which you identify with (gender, social status, ethnicity, sexual orientation, etc.) influence your clinical practice (positively or negatively)? In your opinion, what are the expected characteristics of a doctor’s professional posture and to what extent do they influence your clinical practice? Based on the above considerations, what personal and professional characteristics would you rely on to limit/control your own biases? Generally speaking, what have you learned from this training on reflective practice on gender bias? In your opinion, what tools should be incorporated into your training (undergraduate and post-graduate) to limit gender bias in clinical practice?

The reflection questionnaires were documented and stored on the students’ personal online portfolio. Each questionnaire was read and commented by a gender medicine expert (EG) to provide students with individual feedback. Elements discussed individually in the reflection questionnaire were sometimes anonymously used during the group discussions to trigger further discussions on reflexivity and positionality.

### Data collection

For this study, we extracted the reflection questionnaires documented by all medical students that underwent their internship in the GP outpatient clinic in 2021 (31 students) and in 2022 (45 students) from the PULS portfolio platform. We created one document per student, cumulating their different questionnaires: students who ran their internship over 3 weeks filled 3 questionnaires, while students staying 6 weeks filled 5 questionnaires (twice questionnaire 1, twice questionnaire 2, and once questionnaire 3). The compiled document was recorded using a number and the year (21 or 22), and the student’s name was deleted from the file to ensure anonymity during data analysis. The codebook containing the student’s name and the attributed code was stored on Unisanté’s server in a folder with restricted access to researchers (JS and EG only). Consent to use the reflection questionnaires was obtained via the charter that students accepted when registering on their electronic portfolio, which stated that their anonymised data may be used for research and development purposes. The study was submitted to the Ethics Committee for Research on Human Beings of Canton de Vaud (CER-VD), who stated that ethical approval was not required (Req-2020–00996).

### Data analysis

The analyses were primarily conducted by a female medical historian specialised in gender issues and inequalities in health (first author, FA). The documents were approached as contemporary historical sources and placed in the context of current issues surrounding clinical practices. This enabled establishing a distance from the documents and putting knowledge and practices on gender in medicine into perspective. The coding scheme and themes development were elaborated jointly by FA and a sociologist and epidemiologist working in the field of gender medicine (JS). A thematic analysis framework was applied [15], using MAXQDA2022 software to support the coding work. The architecture from codes to themes was discussed by FA and JS on different occasions to ensure a coherent and comprehensive analysis. As results from previous studies conducted within the Gender reflexive project and published involved only questionnaire 1 data from 2019 [7, 16], we focus here our descriptive analysis on identified gender biases on either newly emerging themes or on themes that were not previously developed and described. We further conducted our analysis on the data from questionnaires 2 and 3, focusing on the elements revealed around positionality and how positionality influenced reflexivity on gender bias in students. The final analysis and elements to be presented in this paper were discussed and finalised between authors (FA, JS and EG). We followed the Consolidated criteria for reporting qualitative research (COREQ) checklist [17], as well as the Sex and Gender Equity in Research (SAGER) guidelines [18] to write up this article.

## Results and discussion

A total of 76 documents were analysed, corresponding to the full population of students who underwent their internship in the GP outpatient clinic in 2021 and 2022. The proportion of female students was slightly higher in the GP outpatient clinic (76%) compared to the proportion found in all medical students at the University of Lausanne (61% in 2022) [19]. This may reflect a larger interest in general practice among female students. The documents were highly heterogeneous in terms of content, but the role played by reflexivity can be noted at first glance: out of 76 documents, 75 identified and described gender biases when reflecting on their own practice or on the practice of their medical supervisor. The group discussions proved useful, particularly for the identification of certain issues such as the risk of cardiovascular disease in women or the underestimation of depression in men, as already highlighted in a previous study [7]. As also revealed from the previous study, an added value of the approach was its integration in clinical practice and the small group discussions that enables benevolent exchanges on potentially sensitive topics. In this new dataset, we found confirmation of these aspects in the writing of medical students. We additionally found that repeating the exercise of identifying gender bias increased the ability for individual reflections, as illustrated by the following quote:

*At first, with the question 'What would have been different if the patient had been male or female?’, I found it difficult to give a personal answer based on lived experience without it sounding like 'nonsensical' thinking. Then, as the sessions progressed, I realised that these thoughts were based on many very real gender biases that gave them meaning. (6_21)*

The group discussions and exchanges between peers enabled putting one's own thoughts into perspective and to acknowledge the shared, social and therefore transversal dimension of stereotypes:

*It's also worth noting that discussing the cases of other fellow students is even more enriching, as it makes us realise that these biases are sometimes immutable and not just based on a personal position. (6_21)*

*Despite all well-wishing thoughts we may have, we all have gender biases (that may even be against our values), so we need to be aware of them and not think we’re immune so that we can always work on them to improve our clinical practice (13_22)*

### Masculinity bias revealing gender bias

We found that the identification of stereotypes in relation to masculinity allowed revealing broader gender biases because these stereotypes question the social dimension of the interplay between gender and health in a direct manner. Indeed, stereotypes related to feminine norms and roles sometimes tended to be limited in analysis by the frame of the difference between sex and gender, driving discussions on the disentanglement between biological and social roots of inequalities. Conversely, gender bias related to masculinity and virility norms and roles did not raise a discussion on biological roots but questioned the societal gender differentiation and hierarchisation process that is reflected in healthcare practice.

*In your opinion, what could be the factors and/or mechanisms that generate these practice/knowledge biases? There is a social factor that is at play in this [reported] bias. With a social construction that has led men to be less expressive or differently demonstrative of their state of mind, in relation to stress or to depression for example. (02_21)*

Another male student identified the same stereotype that influenced, according to him, the management of a patient: *“a man must be strong, never cry, never be depressed and above all never be submissive” (9_21).*

Finally, we found that masculinity bias and how it operates in clinical practice was understood and identified by both male and female students.

### Somatic vs psychogenic bias

Students also reported that gender bias may lead to a difference in clinical reasoning whereby men’s complaints are perceived as somatic, while women’s complaints tend to be suspected as psychogenic. This is illustrated by the following quote of this male student:

*It may be that women have less difficulty expressing their emotions, or even pain, which could give the impression that they are over-playing or are weaker. Men may feel more ashamed to talk about their emotions (men have to be strong in the old mentalities), and anxiety may not be included in the differential diagnosis, since they wouldn't necessarily express it on their own. (70_21)*

One student suggested that patients themselves, through their gendered expression may lead physicians into this bias, as noted by a female student:

*Patients can even transfer these stereotypes to themselves: for example, a woman suffering from chest pain might attribute it to a panic attack or stress, even though it's a heart problem. These views can influence the doctor. (11_22)*

### Gender division of labour and bias in patient history

A paradigmatic example of gender bias in clinical practice is rooted in the gender division of labour. In the Swiss context, traditional gender roles set men in charge of the productive labour and women of reproductive and care work through the well-described phenomenon of the gendered division of labour. Students have reported how, during consultations, men are rarely asked the question of their reproductive and care work because it is assumed that they are (only) involved in productive work.

*The fact that [in this clinical case] it is a man who does the gardening may unconsciously lead us to think that he has done heavy work (e.g. hedge trimming), which favours the musculoskeletal hypothesis. In the other clinical cases, we found that for men we often asked about their profession but not their personal situation, and vice versa for women. This was not the case for my clinical case, but our psychosocial history was very brief and the personal situation was mentioned by the patient himself and not investigated by our questions. Nor did we ask any further questions. (05_22)*

This inequality of treatment in the assessment of the psychosocial situation of patients was quite easily identified by students while identification that the professional situation was not assessed equally in men and women was less noted. In fact, the focus on the family (reproductive) situation of women patients was perceived as pertinent due to its “natural” relation to the reproductive apparatus in women.

### Men and women: different sex lives?

We found that gender bias frequently emerged in a specific step of a consultation in general medicine: sexual anamnesis. Asking questions about sexual practices that may put patients at risk for sexually transmitted infections (STI) – including HIV – is a regular practice when patients consult with a complaint of urinary tract infection or sore throat with fever (a symptom of STI onset).

Students identified and reported that when discussing sexual practices with patients, bias related to gendered representations of sexuality emerged, as illustrated by this quote:

*I think (and we talked about this with the medical intern) that if the patient had been a man, we would have insisted more on the importance of STIs screening, because we think that women tend to have less sex or less libido and we'll take a woman's word for it that she's faithful, which might be more investigated in a man. (7_22)*

Gender bias on sexuality here is related to a perception that women are less active and more trustable in their declarations on sexual practices considered as risky such as having multiple partners. Such stereotypes were also described in the following quote:

*Whereas in women, we can think that they take better care of their sexual health, have fewer partners and more often have protected sex, so the likelihood of them consulting for an STIs is potentially lower. (5_22)*

The consequences of bias are spelled in the first quote above: such representations lead to less investigating risks for STIs meaning that their chances of being correctly and early diagnosed are reduced. In the quote below, investigating risks for STIs did not take place at all and it was in the context of reflexivity work that the question was raised:

*In this case, we didn't ask the patient at all about her sexual relations, so we didn't assess her risk of contracting an STI. Although the status correlated with viral angina, other infectious causes should have been excluded. In a single man, this aspect might have been more easily addressed. (41_21)*

Another student reflected on the fact that STIs may themselves be gendered in their perceptions, as described below:

*A man comes to the clinic with symptoms following unprotected sex with another man. All the STIs requiring screening have been investigated. Despite this, I realised that in the current situation, I was thinking first and foremost of syphilis and HIV, whereas in the case of a woman, I would have mentioned gonorrhoea and chlamydia as the first things to be screened for. It's not that I wouldn't have thought about the other differential diagnosis, but it's as if they were less likely depending on the patient's sex. I surprised myself by thinking like that (12_22).*

The analysis of the documents revealed a general perception of women as asexual persons, or persons with stable, soft and non-risky relations. They also appeared as infantilised individuals who have not yet conquered the right to control their own bodies. One document was particularly interesting, as it raised the issue of contraception management:

*The patient's wish for sterilisation was completely ignored. Although she repeatedly insisted that she did not wish to have any more children, and that she was looking for the most effective method of contraception possible, other options were not mentioned. While the desire for tubal ligation requires special explanation because of the irreversibility of the procedure, the patient should have been informed of all possible options. After the consultation, when I asked the resident why this option had not been proposed to her post-partum, he replied that women often change their minds, and that this possibility should therefore be avoided. With a man, such a desire might have been considered and accepted more readily. (41_21)*

One female student reported that the reflection exercise brought her to realise that she had a “subconscious” bias that women involve more emotions in sexual relations in comparison to men, as described in her reflection questionnaire:

*A 26-year-old patient consulted because he had noticed a purulent discharge when he urinated. He had been on holiday in Dubai for 8 days and had spent the night with an unknown woman. He had protected sex but the condom broke. […] He was released on a course of antibiotics that covered both [gonorrhoea and chlamydia] germs and an appointment was arranged several days later to discuss the results. […] It surprised me a bit when I thought about it, but I think that if the patient had been a woman, I would have been more concerned about her psychosocial state in the same situation, because it seems more “common” to have a sexual adventure on holiday in men compared to women. I would have probably asked more questions about her general mood. I would also have taken a history of her menstruations and the possibility of pregnancy […]. I realise that in the sexual context I make more subconscious conclusions that in other areas of a consultation (08_21)*

To be noted that she did not point out in her reflection document to the fact that the possibility of a pregnancy and the stress potentially caused by it was not assessed with the “real” male patient.

Thus, sexuality of women is mostly thought in mirror to sexuality of men in a heterosexual paradigm: no reference is made to the possibility that the woman could be homosexual. The possibility of homosexuality however emerged for men patients, notably in relation to STIs, hinting towards to tendency to set the “neutral” and standard sexuality in heterosexual men, and perceiving sexuality in women and homosexuality as “other”.

### Positioning oneself: between privilege, empathy and objectivity

When asked to reflect on how the social characteristics which they identified with (gender, social status, ethnicity, sexual orientation, etc.) influence their clinical practice (positively or negatively), most students first described their position through the suggested dimensions. We found that following the group discussions on gender, positioning with regards to gender was easily conducted, and the perceived influence of one’s gender on the capacity to understand, relate and be empathetic with patients of the same or the opposite gender was described, as illustrated by the following quotes:

*Being a white woman from a privileged background I'm aware that I have access to a large number of privileges. However, as a woman I am still confronted with discrimination and sexism, so I may not be aware of all forms of discrimination, but at least some of it. I am trying for my future practice to be as open-minded as possible, as sensitive as possible to bias and stereotypes and as respectful as possible of others. I hope that this will have a positive influence on my clinical practice (13_22).*

A female student identified that she may better relate to female patients’ complaints if she had herself bodily experienced specific situations such as menstrual pain, postpartum depression. In mirror, she suggested that with men, she may not easily relate to some situations:

*Not having lived in a male body, I could have more difficulty in identifying important signs. For example, men conforming with social stereotypes (e.g. men should not show signs of sadness in public, should keep their emotions under control), I could have difficulty in recognising mental distress in a man. My gender thus has positive and negative effects in my clinical practice (05_22).*

A male student suggested that men’s own construction and perception of masculinity may influence their practice:

*Perhaps we tend to project our own social background, ethnicity or sexual orientation onto the patient. If, for example, a doctor who as a child was not allowed to cry because "men don't cry", perhaps when he sees a man cry he will take less account of the patient's emotion and will detach himself from this aspect to investigate elsewhere (09_21).*

When reflecting on position related to social class, we however found that students tended to acknowledge that understanding and relating to lower classes was limited, yet with less insights on concrete issues. In fact, surprisingly, in a context of patient care in public services, students appeared to be at a loss when it came to precariousness. Encouraging them to reflect on their privileged status revealed shortcomings in the training of future practitioners:

*However, having grown up in what I consider a privileged environment (a safe country, with a roof over my head and unrestricted food), I'm fairly uninformed, for example, about people with no health insurance coming to the [clinic]. How do we deal with them? Is there any help available? How can I find resources to take better care of these people, who sometimes have no family in the country, don't speak the language, have no fixed address? (5_22)*

The positioning exercise thus turns into the unveiling of a malaise, almost a feeling of guilt:

*I come from a relatively well-off Swiss family with no socio-economic worries. There are certain difficult experiences that don't echo my own situation at all, and which can overwhelm a person who, like me, knows very little about this kind of situation. What's more, with different visions of health due to culture and education, it can be more difficult to create a bond of trust for a good therapeutic relationship (49_22).*

While several students were able to identify such class difference issue, we however found that they were short in describing how the issue may have an impact on their clinical practice, as well as how it may be overcome. To be noted however that the focus of the reflective exercise was not on class bias, but indeed on gender bias, and this may explain the thinner description of class bias. Other students have described cultural bias with patients coming from diverse geographical and cultural backgrounds, and one student reported his position in relation to religion that may influence her practice:

*Also, because I grew up in a middle-class family, I've never had to deal with existential anxiety, and I don't know how much it can affect physical symptoms. And I didn't grow up very religious either, so sometimes I don't appreciate the importance of religion in the healing process. (06_21)*

The positioning and reflection exercise led students to identify and/or account for their intersectional – largely shared – privileged position. The exercise remained short in engaging students to reflect on what to do with such privilege, especially in students who identified strong privileges: how it can influence a clinical encounter positively or negatively on one hand; and how they may act to minimise the effects of the power dynamics. Based on these observations, we reflected on the need to thematise the notions of power and agency in positionality in the future, in order to bring students to discuss and reflect on how privilege can be positively mobilised or used in clinical encounters.

### Social position or professional neutrality?

Students are looking for strategies to make up for this lack of tools, particularly in the notions of impartiality and neutrality in medicine and empathy on the part of the doctor. In the analysis of positioning, we found that for future doctors, it's not easy to reconcile the notions of social position acquired before their medical studies via socialisation and of professional position that is expected from them, i.e. objectivity, neutrality. This is illustrated by the quotes below:

*"Western culture, religion, the family pattern in which we live, ... I have more the impression that at this stage these are "personal" factors more than professional ones". (70_21)*

*"The doctor must remain neutral, listen to a complaint, treat an illness and generally take care of a patient. Whether the patient is a woman, a man, white, black, Muslim, Jew, heterosexual or homosexual". (70_21)*

Objectivity would thus be in sharp contrast to positioning. Positioning would lead to an unveiling of the self and the other, which would prevent the student from maintaining a neutral but empathetic listening position at the same time.

*"Above all, the doctor must maintain a non-judgmental and benevolent attitude towards the patient. Even in the event of moral disagreement, the doctor must put his or her opinion aside as best he or she can, so as not to jeopardize the patient's care. (69_21)*

One student acknowledges that his/her attitude may be positively biased when patients share a similar background in terms of language, and negatively biased when patients are not aligned, in this case in relation to mental health:

*"I think I'll be positively influenced if I see someone from a similar social background to me (e.g. someone who speaks French, as it'll be easier to communicate). I don't think gender, ethnicity or sexual orientation influence my practice (positively or negatively). On the other hand, unfortunately, I think I'm negatively influenced by people with psychiatric pathologies/psychic distress, etc.... I have the impression that since I understand them less, I find it harder to be empathetic and "believe" their complaints. (72_21)*

The inherent tension between the quest for objectivity and use of scientific evidence and the reality of social encounters in clinical practice has been articulated as a science/culture or science/art dichotomy in the medical humanities [20, 21]. Carmel [22] has conceptualised medical practice as a “craft” activity that brings together different types of knowledge, skills and practical judgements of physicians and the material world, i.e. clinicians’ and patients’ bodies and technological artefacts. In the field of gender medicine, the task is to better integrate the cultural dimension of gender into biomedical research and clinical practice in order to account for the ways in which socially constructed gender roles and norms modulate the health and well-being of individuals through gendered differences in exposure, gendered health-related behaviours and gendered impacts on accessing care [23]. The exercise of reflexivity and positioning aims firstly to identify gender bias in clinical interactions and then to improve the clinical “crafting” by minimising the effects of implicit bias that occur in the material world, i.e., in a consultation where the social representations of clinicians and patients interact. The field of gender medicine also aligns with what Whitehead and Kuper have called “a false dichotomy” in the currency of medicine as an art and a science. The scholars point out that the science part is not seen as problematic and suggest that “the construction of science as facts and evidence is an oversimplification that must also be considered by medical educators” [24]. Building on the extensive work of gender studies, feminist scholars have questioned the situated and gendered production of medical knowledge, beginning by describing the erroneous medical assertions of “natural” sex differences that have justified a series of political claims (e.g. banning women from voting or practicing medicine) [25]. More recently, scholars have scrutinised the sex dimension – measured mainly by the male/female variable – which has been oversimplified as ontological and biological evidence in biomedical research and little considered until now [26, 27].

### Being underprivileged as a privilege?

Having a non-conventional or non-privileged background was perceived as an asset to rely to the situation of some patients, as reported by this female student:

*Perhaps I'm more empathetic and compassionate than the average man, perhaps not necessarily genetically but through education or social expectations. [...] As a German, with non-French speaking patients I often have the feeling that I have an advantage because I know how to speak "simple" French a bit better [...] I also think I'm influenced rather positively by my social background because I didn't grow up in the academic sphere but in a rather rough neighbourhood. Now I feel that sometimes it helps me to understand a bit better patients that come from more difficult social environments and to feel more at ease. (08_21)*

*I feel that being a woman helps me have a cultural gaze that is more empathetic, but also helps me understand what discrimination means, and so to be more attentive to discrimination (07_22).*

This phenomenon of positionality being facilitated when individuals occupy unprivileged facets was described by Zhou in her article titled “Underprivilege as a privilege” [28]. This was also reported by Blalock et al. who studied how women medical students navigated the (sexist) world of medical schools in the USA to become doctors. Analysing their qualitative longitudinal data, they indeed found that “these students are acutely aware of their positionality and intersectional identities, reflecting on their roles as women, their body size and shape, their ethnicity, and religious identities, and the implications this has on their interactions with patients” [29].

## Conclusion

In 2009 in this journal, Risberg, Johansson and Hamberg proposed a theoretical model for analysing gender bias in medicine. They concluded that teaching medical students about facts on biological differences and/or evidence of bias would not reduce bias, because bias is “caused by gendered stereotypes or by unawareness of health problems and discrimination associated with gender inequity”. They added a suggestion to implement “consciousness-raising activities and continuous reflections on gender attitudes among students, teachers, researchers and decision-makers”[3]. Our study confirms previous findings that implementing a gender bias reflexivity module with medical students during their internship has provided positive results in terms of identification of gender biases [7]. Adding elements of positionality and repeated reflections on gender bias has enabled uncovering a tension experienced by students between the process of *becoming doctors* – i.e. the enculturation into the medical world and its injunction of neutrality and objectivity – and the exercise of positioning in a reality of diversity and power that are rooted in social and subjective worlds. We conclude that such tension should be explicitly discussed with students because it is constitutive of medical practice. The existence and influence of power dynamics and social interactions need to be re-injected and discussed during medical training, in order to better reflect and prepare for (future) medical practice. In other words, the accompaniment of enculturation into medical doctors should encompass and encourage inclusion of social and gendered dimensions, because they can not be simply erased from reality. Thus, reflexivity is a major tool of medical education; it should not be implemented in isolation but rather fully integrated to address gender bias, and other intersectional biases. More research is however needed to understand if and how such sensitisation will carry long-term effects in the future medical practice of students.

## Data Availability

The datasets analysed during the current study are available from the corresponding author on reasonable request.

## Abbreviations

EDI: Equality, Diversity and Inclusion
CER-VD: Ethics Committee for Research on Human Beings of Canton de Vaud
GP: General Practice
HIV: Human Immunodeficiency Viruses
STI: Sexually Transmitted Infections
